# 

**Published:** 1884-03-20

**Authors:** 


### TABLE OF CONTENTS.

ORIGINAL AND SELECTED ARTICLES:

The Relation of Errors of Refraction and Accommodation to Defective Sight, and to Some Nervous Disturbances, by A. G. Hobbs, M.D., of Southern Medical College, Atlanta, Georgia, 81; Case of Man vs. Monkeys, Dogs et. al., by E. Van Goidtsnoven, M.D., of Georgia, 88; Biliary Calculi, by C. S. Webb, M.D., of Virginia, 93; Orchitis, by H. M. Lawson, M.D., of Georgia, 95; Hepatic Dyspepsia, 95; Some Points concerning Sanguis Bovinus Exsiccatus (Desiccated Blood), 97; Baltimore Medical Association, 99.

ABSTRACTS AND GLEANINGS:

Homeopathy in the British Medical Association; Treatment of Measles, 101; Experimental Investigation into the Protective Power of Attenuated Tuberculous Material, 102; Radical Cure of Hernia, 103, Absorbent Cotton as a Dressing; What is a Couvreuse? 104; Injection of Morphia for Strangulated Hernia; Salicylic Acid in Cerebro-Spinal Meningitis; Glycerine after Bathing, 105; The Cholera Germ; International Medical Congress; Good Advice, 106; Hippurate of Soda, 107; Antral Abscess; Dr. B. W. Richardson on the Morphia Habit and its Treatment, 108; Anæsthesia by a mixture of Morphia, Atropia and Chloroform, 109; Effects of Strong Light upon the Eye; Succinate of Iron in Biliary Colic; Gelsemium for After-Pains; Iodoform in Ophthalmic Therapeutics, 110.

SCIENTIFIC ITEMS:

Metallic Cloth; Enormous Growth of Railway Interests; On the Possibility of making a Cadaver Disappear Entirely by the use of ordinary Sulphuric Acid, 111; The Earth More Rigid than Steel; Confirmation of Professor Young's Explanation of the Atmospheric Glow; The Beautiful Snow, 112.

PRACTICAL NOTES AND FORMULÆ.

A Reliable Tænifuge; Oleic Acid for Itching; For Dyspepsia with Constipation and Piles; Iodide of Ethyl; Malarial Poisoning, 113; Treatment of Chapped Hands and Frosted Feet; Flatulent Dyspepsia; Local Anæsthesia; Dr. Goodell's Mixture of the Four Chlorides, 114.

EDITORIALS AND MISCELLANEOUS.

Commencement Exercises of the Southern Medical College, Session 1883-1884, 115; Editorial Notices, 118: The Society Troubles in New York—A New Organization; Books and Pamphlets Received, 119; Receipted; Special Notices, 120.

